# Learning Outcomes of Immersive Technologies in Health Care Student Education: Systematic Review of the Literature

**DOI:** 10.2196/30082

**Published:** 2022-02-01

**Authors:** Grace V Ryan, Shauna Callaghan, Anthony Rafferty, Mary F Higgins, Eleni Mangina, Fionnuala McAuliffe

**Affiliations:** 1 Perinatal Research Centre Obstetrics and Gynaecology School of Medicine, University College Dublin Dublin Ireland; 2 School of Computer Science University College Dublin Dublin Ireland

**Keywords:** Virtual Reality, Augmented Reality, Mixed Reality, Learning Outcomes, Medical Education, Nursing Education, Midwifery Education, Systematic Review

## Abstract

**Background:**

There is a lack of evidence in the literature regarding the learning outcomes of immersive technologies as educational tools for teaching university-level health care students.

**Objective:**

The aim of this review is to assess the learning outcomes of immersive technologies compared with traditional learning modalities with regard to knowledge and the participants’ learning experience in medical, midwifery, and nursing preclinical university education.

**Methods:**

A systematic review was conducted according to the Cochrane Collaboration guidelines. Randomized controlled trials comparing traditional learning methods with virtual, augmented, or mixed reality for the education of medicine, nursing, or midwifery students were evaluated. The identified studies were screened by 2 authors independently. Disagreements were discussed with a third reviewer. The quality of evidence was assessed using the Medical Education Research Study Quality Instrument (MERSQI). The review protocol was registered with PROSPERO (International Prospective Register of Systematic Reviews) in April 2020.

**Results:**

Of 15,627 studies, 29 (0.19%) randomized controlled trials (N=2722 students) were included and evaluated using the MERSQI tool. Knowledge gain was found to be equal when immersive technologies were compared with traditional learning modalities; however, the learning experience increased with immersive technologies. The mean MERSQI score was 12.64 (SD 1.6), the median was 12.50, and the mode was 13.50. Immersive technology was predominantly used to teach clinical skills (15/29, 52%), and virtual reality (22/29, 76%) was the most commonly used form of immersive technology. Knowledge was the primary outcome in 97% (28/29) of studies. Approximately 66% (19/29) of studies used validated instruments and scales to assess secondary learning outcomes, including satisfaction, self-efficacy, engagement, and perceptions of the learning experience. Of the 29 studies, 19 (66%) included medical students (1706/2722, 62.67%), 8 (28%) included nursing students (727/2722, 26.71%), and 2 (7%) included both medical and nursing students (289/2722, 10.62%). There were no studies involving midwifery students. The studies were based on the following disciplines: anatomy, basic clinical skills and history-taking skills, neurology, respiratory medicine, acute medicine, dermatology, communication skills, internal medicine, and emergency medicine.

**Conclusions:**

Virtual, augmented, and mixed reality play an important role in the education of preclinical medical and nursing university students. When compared with traditional educational modalities, the learning gain is equal with immersive technologies. Learning outcomes such as student satisfaction, self-efficacy, and engagement all increase with the use of immersive technology, suggesting that it is an optimal tool for education.

## Introduction

### Background

Educational technology is changing the way in which we learn today, and its purpose is to ultimately improve education [1,2]. The addition of educational technology to a curriculum needs to be developed and guided by informed, evidence-based research. Educational technology includes instructional software such as virtual reality (VR), augmented reality (AR), and mixed reality (MR), known collectively as immersive technology [3]. Immersive technologies should be built around effective teaching methods that provide an appropriate learning method and learning outcome [4]. Immersive technologies are thought to provide pedagogy based on the constructivist theory and experiential learning, creating an environment that aids visual learners and enables students to learn by doing, develop creativity, and increase understanding of invisible concepts [5]. The Association for Medical Education in Europe has previously published guidance on e-learning in medical education: “Designs for effective medical e-learning, therefore, need to mirror the dynamics and details of real-world practice as well as affording effective learning opportunities” [6].

Immersive technologies are defined as devices that provide sensory stimuli to provide a sense of realism and immersion in the interactions with the computer-generated world [7]. VR is a technology that allows the user to explore and manipulate computer-generated real or artificial 3D multimedia sensory environments in real time. It dates back to 1956, when Morton Heilig, a cinematographer, developed *the Sensorama*, a display box first used for background scenes in the Hollywood motion picture industry. This was the first head-mounted display to be developed. In the mid-1960s, Ivan Sutherland, an American Computer Scientist, went on to develop the concepts of VR further. He described *The Ultimate Display*, a VR system that could simulate reality [8], and his paper described core concepts that are the foundation of VR use today. Owing to the heterogeneity of the terminology used to describe VR, we can characterize VR as a “collection of hardware such as Personal Computer (PC), HMDs and tracking sensors, as well as software to deliver an immersive experience” [9]. In comparison, AR is an interactive experience of a real-world environment where the objects that reside in the real world are *augmented* by computer-generated perceptual information. Historically, the development of AR started in the 1960s; however, the term *AR* was not established until 1990. Although VR and AR share many technical aspects, the main difference is that AR does not construct a fully artificial environment and simply overlays computer-generated images onto images of the real world. Therefore, it uses machines that allow a physical view of the surrounding environment to be visible but enhanced with virtual images [10]. Finally, MR is the merging of real and virtual worlds to produce new environments and visualizations where physical and digital objects coexist and interact in real time [11].

### Objective

To date, there has been a multitude of publications detailing the development and implementation of immersive tools, in addition to demonstrating the benefits of VR, AR and MR technology in medical, nursing and midwifery education [12-17]. This technology is thought to provide increased engagement and understanding during learning coupled with feedback mechanisms and design capabilities of varying difficulty levels [5]. In addition, it facilitates practice without the risk of human harm and also helps build professional skills and teamwork [18,19]. However, there is a paucity of evidence on the learning outcomes of these innovative educational tools.

As outlined by the Digital Health Education Collaboration, there is a need for a strong evidence base to guide the development of immersive educational tools so that the learning goals and outcomes are in line with national and international standards [20]. There have been an increasing number of systematic reviews documenting the use, application, and effectiveness of VR, AR, and MR in an effort to establish an evidence-based network of research for use in medical education. However, the results have been mixed; including a systematic review that looked at the effectiveness of AR in medical education which found that there was insufficient evidence to recommend its implementation into the curriculum. Similarly, another review looked at serious games used in medical education and found that the evidence was moderate to support the use of immersive technology, stating that it should not replace traditional learning tools [21-25]. Immersive technologies are used mainly as educational tools for complex topics such as anatomy and embryology and are thought to enhance the learning experience [26,27]. Are VR, AR, and MR as effective in delivering knowledge as well as an enhanced learning experience in comparison with traditional teaching tools such as 2D didactic presentations?

Therefore, the aim of this systematic review is to assess the learning outcomes of VR, AR, and MR across 3 health care student disciplines—medicine, nursing, and midwifery education—compared with traditional learning modalities. The learning outcomes include knowledge, skill development, and the learning perceptions of students, including satisfaction and self-confidence in learning along with engagement and motivational factors.

## Methods

### Purpose and Protocol

A systematic review of the available scientific literature was conducted to assess the learning outcomes associated with the application of VR, AR, and MR as educational tools compared with traditional learning modalities for medical, nursing, and midwifery students in preclinical university education. The review protocol was registered with PROSPERO (International Prospective Register of Systematic Reviews) in April 2020 (CRD42020154598). The search results were reported according to the PRISMA (Preferred Reporting Items for Systematic Reviews and Meta-Analyses) guidelines [28] and the Cochrane Collaboration guidelines [29].

### Eligibility Criteria

The eligibility criteria were based on the Population, Intervention, Comparison, and Outcomes criteria. The population selected for this review included preclinical university students enrolled in three educational disciplines: medicine, nursing, or midwifery courses only. Randomized controlled trials (RCTs) that implemented VR, AR, or MR technology in comparison with a control method were included. Owing to the heterogeneity of the definitions surrounding VR applications, we restricted inclusion to interactive 3D models requiring a headset, virtual patients (VPs), or VR learning environments. The primary outcomes included knowledge and reference to the learning experience, which involved engagement, satisfaction, and perceived learning experience. Only studies published in English were included.

### Search Strategy

A large-scale search was undertaken because of the wide use of the various terminology to describe VR, AR, and MR and the technology surrounding their use in health care student education. The following method was used to identify empirical studies for inclusion in the systematic review. We conducted a comprehensive computerized database search of full-text articles published in English. Only RCTs assessing learning outcomes using VR, AR, or MR technologies in comparison with traditional learning models were included. The reason for this was that we wanted to review the learning outcomes of immersive technologies compared with traditional learning outcomes, including knowledge and learner experience. The fundamental study design of an RCT requires a control and an intervention group; therefore, we selected these types of studies for this review. Searches were conducted with predefined search terms (Multimedia Appendix 1) using the following electronic databases: PubMed, Embase, Web of Science, CINAHL, and ERIC. Medical Subject Headings terms included *virtual reality*, *augmented reality*, *educational technology*, *imaging*, *three dimensional*, *education*, and *teaching materials*. Search terms were connected using Boolean operators *AND* and *OR* to capture all relevant article suggestions. The latest search was conducted on March 8, 2021.

**Figure 1:**
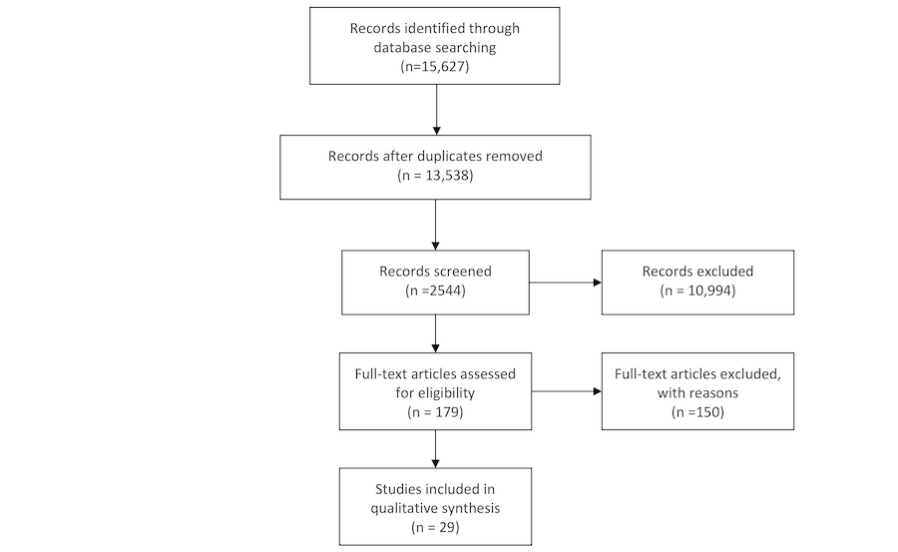
PRISMA (Preferred Reporting Items for Systematic Reviews and Meta-Analyses) 2020 flow diagram adapted for this study.

Databases were downloaded to EndNote reference manager software (Clarivate Analytics), which recorded citations and identified duplicates. A spreadsheet was created to record decisions and comments. Screening of articles was conducted by 2 researchers (GR and SC) in an unblinded, standardized approach (independently and in parallel). Titles and abstracts of studies sourced from electronic databases were reviewed according to the inclusion and exclusion criteria previously described. Subsequently, full texts of the included articles from the initial screening process were reviewed for eligibility according to the described inclusion and exclusion criteria. Differences of opinion were resolved through conversations between the reviewers. A schematic stepwise algorithm for the search strategy is shown in Figure 1.

### Data Collection

Microsoft Excel was used to build a data extraction form, which was divided into three categories: (1) study identification, (2) analysis of learning outcomes, and (3) study design. The first section included bibliographic information, the country of origin of the study, and a demographic description of the participants. The second section examined learning outcomes related to teaching strategies, relationships between technologies, and learning objectives. The third section evaluated the methodological quality of the study design.

### Study Quality Assessment

We used the Medical Education Research Study Quality Instrument (MERSQI) to evaluate the study design of the RCTs [30]. The MERSQI is divided into several domains, including evaluation of study design, sampling, data type, validity, data analysis, and outcomes. The learning outcomes are based on the hierarchy of educational outcomes by Kirkpatrick and Kirkpatrick [31], which adopts a constructional framework using a 4-level model for evaluating educational effectiveness. The first level describes the participants’ perception of the learning experience; knowledge, skills, and attitudinal change are assessed in the second level; changes in behavior are evaluated in the third level; and changes in health care or patient outcomes are evaluated in the fourth level.

### Data Analysis

A narrative review of the results reported in the included studies on learning outcomes was conducted. The data in the final included studies did not allow for a formal meta-analysis as the studies were not sufficiently homogenous, given the stated research question and the use of different technologies and educational topics.

## Results

### Study Selection

We identified 15,627 articles from the primary database search. After duplicates were removed, there were 86.63% (13,538/15,627) of articles left for abstract review. Abstracts were screened and, of those 13,538 articles, 179 (1.32%) remained for a full paper review. Of those 179 articles, 150 (83.8%) were excluded, leaving 29 (16.2%) full papers for study inclusion. Details of the study selection process are displayed in Figure 1. In total, 29 RCT studies (N=2722) were included in this review. All studies were conducted in the past 10 years, with most studies (18/29, 62%) published within the past 3 years.

### Study Designs

In total, 2722 students participated in the 29 RCTs. Of the 29 articles, 19 (66%) included medical students (1706/2722, 62.67%), 8 (28%) included nursing students (727/2722, 26.71%), and none of the studies involved midwifery students. Approximately 7% (2/29) of studies included both medical and nursing students (289/2722, 10.62%). The following disciplines were used to test the immersive technologies: anatomy, basic clinical and history-taking skills, neurology, respiratory medicine, acute medicine, dermatology, communication skills, internal medicine, and emergency medicine. A full list of the RCTs, basic demographic details, and immersive technology applications included in this review is outlined in Table 1.

**Table 1:** Randomized controlled trials included in this review of immersive educational tools.

Author	Setting	Application detail	Sample size, N	Purpose	Outcome
Seifert et al [32]	Germany	VP^a^ cases (Moodle learning management system)	40	VP—basic clinical skills	Similar levels of long-term knowledge gained; participants assessed the learning experience and the comprehensibility of the seminars as either *very good* or *good*
Wang et al [33]	New Zealand	3D visualizer software (preloaded 3D hologram) on Microsoft HoloLens device	52	Anatomy teaching	There was no difference in knowledge acquisition between groups; only MR^b^ group demonstrated higher retention in nominal and spatial types of information; increased engagement in 3DM^c^ and MR group
Rossler et al [34]	United States	Virtual Electrosurgery Skill Trainer developed by the National Institutes of Health	20	Fire safety knowledge	No differences in knowledge; intervention group participants were noted to meet performance criteria for their assigned role in their perioperative team
Lombardi et al [35]	United States	Virtual heart activities using physiology software programs (Practice Anatomy Lab, Pearson Education, and Interactive Physiology)	29	Anatomy teaching	Plastic model group achieved significantly higher overall scores on initial and follow-up exams; attitude surveys demonstrated a higher preference for organ dissection
Padilha et al [36]	Portugal	Body Interact (simulation with VPs)	42	Respiratory medicine	Improved knowledge and higher levels of learning satisfaction in the intervention group; no statistically significant differences in self-efficacy perceptions
Blanie et al [37]	France	VP cases—LabForSIMS (simulation center) and a software designer (Interaction Healthcare)	146	Basic clinical skills	No significant educational difference was found; satisfaction and motivation were found to be greater with the use of SG^d^
Liaw et al [38]	Singapore	VP simulation—eRAPIDS, developed at the National University of Singapore	57	Clinical deterioration	No difference in knowledge acquisition; VP was rated positively
Menzel et al [39]	United States	Second Life (Linden Lab) virtual simulation environment (WALD^e^ Island)	51	Cultural attitudes	No statistically significant differences between the learning formats
Gananasegaram et al [40]	Canada	Campbell’s 3DM of the inner ear—publicly available data sets displayed on Microsoft HoloLens	29	Anatomy teaching	No difference in knowledge acquisition; HG^f^ group rated higher for overall effectiveness, ability to convey spatial relationships, and learner engagement and motivation
Liaw et al [41]	Singapore	VR^g^ (no details)	198	VP to teach MDT^h^ rounds	Increased levels of self-efficacy and attitudes toward interprofessional team care
Moro et al [42]	Australia	Microsoft HoloLens, 3D Studio Max (Autodesk Inc), Unity 3D (Unity Technologies), Vuforia v5 plug-in for Unity (PTC Inc), Samsung Galaxy Tab 3 (Samsung Electronics), Visual Studio v2019	40	Physiology and brain anatomy	No difference in knowledge test scores; significant increase in dizziness using the HoloLens
Stepan et al [26]	United States	VR model of brain anatomy—brain CT^i^ scans and MRIs^j^, Surgical Theater, Oculus Rift VR system (Oculus VR)	66	Cerebral anatomy	No difference in anatomy knowledge; VR group found learning experience to be significantly more engaging, enjoyable, useful, and motivating
Hu et al [43]	Taiwan	Anatomy Master module of Medical Holodeck	101	Anatomy teaching	Significant improvement in ultrasound task performance and ultrasonographic image identification MCQ^k^ tests in the VR group
Engum et al [44]	United States	CathSim Intravenous Training System (CathSim) developed by HT Medical (Immersion)	93	Intravenous catheter training	Significant improvement in cognitive gains, student satisfaction, and documentation of the procedure with the traditional laboratory group compared with the computer catheter simulator group
Berg et al [45]	Norway	VR application developed by the authors with hired help for programming (Unity 2018.30f2) and video of the VR features	289	ABCDE basic resuscitation skills	Noninferiority of learning modality; more students in VR group reported liking the way they practiced and that it was a good way to learn; VR group scored high on the System Usability Scale
Kiesewetter et al [46]	Germany	VR learning environment CASUS	142	VP to teach clinical skills	Case formats with a VP did not affect knowledge gain or diagnostic accuracy [46]
Schoeb et al [47]	Germany	Instructions for catheterization displayed on Microsoft HoloLens	164	Catheter training	MR group had significantly better learning outcomes [47]
Noll et al [48]	Germany	AR^l^ mobile app, iPhone operating system (iOS, Apple Inc)–based app mArble Derma (m-ARBLE-dermatology)	44	Dermatological teaching	No difference in outcomes between groups [48]
Liaw et al [49]	Singapore	3D virtual hospital developed—CREATIVE	120	Interprofessional skill training	No difference between groups in communication performance scores [49]
Ienghong et al [50]	Thailand	3D USS^m^ images played on the downloaded phone app and AR	46	Emergency ultrasound skills	Better performance scores in VR flash card group [50]
Sobocan et al [51]	Slovenia	VP—no detail	34	Internal medicine skills	No difference in exam performance between groups
Kockro et al [52]	Switzerland	Virtual 3DM developed from MRI and CT scans and DextroBeam system (Bracco Advanced Medical Technologies)	169	Neuroanatomy	There were no significant differences in knowledge scores; participants rated the 3D method as superior to 2D teaching methods in four domains: spatial understanding, application in future anatomy classes, effectiveness, and enjoyableness [52]
Nickel et al [53]	Germany	Computer-based TM^n^ developed using the open-source Medical Imaging Interaction Toolkit software developed by the German Cancer Research Center	410	Liver anatomy	3D group had higher scores, and participants had a preference for 3D training [53]
Berg et al [54]	Norway	ABCDE resuscitation application developed with help from hired programmers for Unity using Oculus Quest software (Oculus)	289	VP to teach clinical skills	Group self-practice of the ABCDE approach in multiplayer, immersive, interactive VR application was noninferior to practice with physical equipment [54]
Bogomolva et al [55]	The Netherlands	DynamicAnatomy AR application developed at the Department of Anatomy and Embryology at the Leiden University Medical Centre for Innovation	60	Anatomy teaching	No significant differences in knowledge scores [55]
O’Rourke et al [56]	United States	VP model simulation developed with a real patient in real time	60	Clinical skill—breaking bad news	No significant between-group differences on the POMS^o^ 2 or salivary cortisol concentration following the SP^p^ interaction; students had similar emotional and behavioral responses when delivering bad news to SP or vSP^q^ [56]
Du et al [57]	Taiwan	3DMs—Autodesk 3DS Max software (Autodesk Media and Entertainment) and Unity 3D developed into a VR gaming system for HTC Vive	18	Anatomy teaching	No significant differences in MCQ scores between groups; VR groups scored highly on the interest, competence, and importance subscales of the IMI^r^; both VR groups considered the system to be fun and beneficial to their learning; MP^s^ group reported higher stress levels
Maresky et al [12]	Canada	Using TeraRecon (TeraRecon, Inc), Slicer, and The Body VR: Anatomy Viewer private beta version (The Body VR LLC) together with software provided by Sharecare VR (Sharecare Reality Lab)	42	Cardiac anatomy	VR group scored higher on knowledge
Issleib et al [58]	Germany	VR software developed in cooperation between the University of Hamburg and VIREED^t^—VR-BLS^u^ course (using the Laerdal (mannequin)	160	Resuscitation training	Classic BLS training with a seminar and training sessions seemed superior to VR in teaching technical skills [58]

^a^VP: virtual patient.

^b^MR: mixed reality.

^c^3DM: 3D model.

^d^SG: simulation by gaming.

^e^WALD Island named for Lillian Wald, a public health nursing pioneer.

^f^HG: holographic.

^g^VR: virtual reality.

^h^MDT: multidisciplinary team.

^i^CT: computed tomography.

^j^MRI: magnetic resonance imaging.

^k^MCQ: multiple-choice question.

^l^AR: augmented reality.

^m^USS: ultrasound scan.

^n^TM: teaching module.

^o^POMS: Profile of Mood States.

^p^SP: simulated patient.

^q^vSP: virtual simulated patient.

^r^IMI: Intrinsic Motivation Inventory.

^s^MP: multiple-player.

^t^VIREED: Virtual Reality Education (medical virtual reality education platform).

^u^BLS: basic life support.

### Study Characteristics

Approximately 76% (22/29) of authors used VR applications, which included virtual simulation scenarios and VPs. Approximately 17% (5/29) of articles used AR applications, which involved using the Microsoft HoloLens headset and mobile phone apps. There were 7% (2/29) of studies that used MR.

Of the 29 articles retrieved, 28 (97%) were from high-income countries and 1 (3%) was from an upper–middle-income country. Most studies originated in the United States (6/29, 21%) and Germany (6/29, 21%), followed by Singapore (3/29, 10%), Canada (2/29, 7%), Norway (2/29, 7%), Taiwan (2/29, 7%), Australia (1/29, 3%), France (1/29, 3%), the Netherlands (1/29, 3%), New Zealand (1/29, 3%), Portugal (1/29, 3%), Slovenia (1/29, 3%), Switzerland (1/29, 3%), Thailand (1/29, 3%), and Turkey (1/29, 3%).

### Primary and Secondary Learning Outcomes

Learning outcomes were reported in all studies, including outcomes for both knowledge and the participants’ learning experience. Knowledge was assessed via multiple-choice question tests, single best answer tests, general clinical knowledge tests, open-ended style tests, or objective structured clinical examinations. Of the 29 studies, 12 (41%) studies evaluated knowledge using multiple-choice question tests. Approximately 31% (9/29) of studies evaluated knowledge via general clinical knowledge–based tests that used a variety of validated questionnaires such as the Maastricht Assessment of Simulated Patients questionnaire and the Attitudes toward Poverty scale questionnaire [39,56]. Approximately 10% (3/29) of studies assessed knowledge using open-ended style exam questions. Approximately 3% (1/29) of studies evaluated knowledge via an objective structured clinical examination–based exam. Of the 29 studies, 1 (3%) evaluated knowledge via a single best answer test, 1 (3%) did so via interprofessional skill assessment, and 1 (3%) did not specify the type of evaluation test. Approximately 90% (26/29) of studies reported on satisfaction, attitudes, perceptions, opinions, and general facts regarding the technology used. The learning experience was evaluated in various ways, including validated and nonvalidated instruments on satisfaction, engagement, and perceived learning outcomes. Approximately 3% (1/29) of studies used psychometric testing to evaluate the learning experience [33]. Another study measured salivary cortisol levels before and after the intervention to evaluate whether delivering bad news via a real simulated patient or a virtual simulated patient evoked the same psychological stress [56]. Approximately 66% (19/29) of studies used validated scales to assess the learning experience, namely, the Intrinsic Motivation Inventory [57], General Self-Efficacy Scale, Learner Satisfaction with Simulation Scale [36], and Diagnostic Thinking Inventory [51]. A full list of the validated scales used in the RCTs in this review is provided in Multimedia Appendix 2 [26,33,34,36-38,41,42,45-49,51,54-58]. Approximately 7% (2/29) of studies reported on behaviors as an outcome, and none of the studies reported patient or health care outcomes [51,56]. Only 3% (1/29) of studies reported on adverse health outcomes as part of their secondary outcomes [42].

### Study Quality Assessment

The MERSQI scale scores ranged from 10 to 15, with a mean score of 12.64 (SD 1.6). The median was 12.5, and the mode was 13.5. The mean (SD) domain and item scores are illustrated in Multimedia Appendix 3. The mean domain scores were highest for study design (mean 1, SD 0), data analysis (mean 0.7, SD 0.46), and outcomes (mean 0.6, SD 0.21). The lowest-scoring domains included type of data (mean 0.5, SD 0), sampling (mean 0.3, SD 0.15), and validity of the evaluation instrument (mean 0.3, SD 0.27). All articles used an RCT (29/29, 100%) study design, which meant that all studies obtained the highest score in this domain.

Of the 29 studies, 1 (3%) was a double-center RCT, and the remaining 28 (97%) were conducted at a single site. In relation to participant response rate, 93% (27/29) of the studies had a high response rate of >75%.

The authors used appropriate statistical analysis in all studies according to the MERSQI [59]. In relation to the validity of the evaluation instrument, 66% (19/29) of studies used validated instruments and scales to assess learning outcomes, including satisfaction, self-efficacy, engagement, and perceptions of the learning experience.

Finally, according to classification using the criteria by Kirkpatrick [31] in the MERSQI scale, 97% (28/29) of studies assessed knowledge as the primary outcome, and 62% (18/29) of studies used a pre- and postlearning experience knowledge assessment.

## Discussion

### Principal Findings

The aim of this systematic review was to assess the current educational role of immersive technology in medical, midwifery, and nursing education at the university level compared with traditional learning modalities. Second, the review evaluated the associated learning outcomes of VR, AR, and MR and how they were evaluated. A total of 29 RCTs were selected for this review. The main findings of this study indicate that knowledge gain is equal when using VR, AR, or MR in health care student education compared with traditional learning modalities. In addition, VR, AR, and MR provide an enhanced learning experience based on the secondary outcomes of the studies included in this review [12,26,33,36,38,40,45]. This supports the current evidence that immersive educational technology is a useful and valuable learning tool in medical and nursing preclinical university education. The most common form of immersive technology used was VR. The favored use of VR may be due in part to the widespread availability of cost-effective 3D software and web-based material for the development of anatomy tools [14,60]. Comparators were present in every study and ranged from 2D didactic presentations to textbooks. All the studies retrieved involved medical and nursing students in preclinical university education. Interestingly, there were no studies involving midwifery students. Although there are studies involving midwifery students and immersive technologies in the literature, this review found no RCTs within this domain.

The MERSQI tool provided a standardized approach to evaluating the methodological quality of the studies included in this review [59], which resulted in a moderate level of evidence for these studies. The MERSQI tool also allowed us to classify the learning outcomes of the RCTs clearly, with knowledge identified as the most common primary outcome. Secondary outcomes were identified and included attitudes and opinions related to the learner experience (ie, satisfaction, self-efficacy, engagement, and perceptions of the learning experience). All studies were conducted in upper–middle-income or high-income countries, which may be because of the availability of more funding to support the purchase of such equipment. The articles identified for this review were published within the past 10 years, with most (19/29, 66%) being published in the past 3 years. This highlights the recent rise in interest in immersive technologies for use in preclinical university medical and nursing education. The International Data Corporation has forecasted the shipment of 36.7 million VR headset units by 2023 [61].

Anatomy was the most common topic taught with immersive technologies, which may be because of the ability of immersive technologies to enhance the understanding of complex body processes that cannot be visualized [24]. Subjects such as anatomy and embryology require students to translate 2D images into 3D concepts, which can be a cognitive challenge for those who have difficulty visualizing this transformation of images [62,63]. Students may also mentally rotate complex structures inaccurately, leaving the mind to fill the gaps in any missing structures [64]. The introduction of immersive technology as an educational tool could automatically standardize this process for students and enhance the understanding of certain subjects such as anatomy and embryology. Similarly, midwifery education requires an understanding of complex concepts, such as fetal development in the womb. Therefore, this would be an important area for future research comparing traditional learning tools with immersive technologies.

VPs were developed in 41% (12/29) of the studies in this review. VPs play a key role in learning basic clinical skills for both nursing and medical education. In addition, they create an environment for repeated practice in a safe space without harm to patients [65,66]. Traditionally, clinical education relies on practicing diagnostic, therapeutic, and procedural skills on live patients. This can be difficult to balance on a day-to-day basis because of the fast-paced nature of medicine, time constraints for clinical teaching in busy wards, and competition between students [67]. In addition, over the past year, the experience of the global COVID-19 pandemic has vastly reduced access to bedside teaching for nursing, midwifery, and medical students [68]. Therefore, the availability and development of VPs and immersive learning environments can play a key role in the future as an adjunct to developing clinical skills for students at all levels and at any time [61].

The primary outcome in most studies (28/29, 97%) was knowledge. This review demonstrated that immersive technology is equally effective in knowledge gained by the student and, in some studies, reflects a higher level of knowledge retention. Regarding secondary outcomes concerning the learning experience, all studies reported overall positivity and higher satisfaction in learning, self-efficacy, and engagement with immersive technologies. Moreover, this review revealed the heterogeneity of tools and instruments used to evaluate secondary learning outcomes such as student satisfaction, self-efficacy, engagement, and learning perceptions. Several studies developed their own Likert-style assessment scales, whereas others used and adapted previously developed and validated scales for the assessment of secondary learning outcomes. According to the Association for Medical Education in Europe, “assessment tools selected should be valid, reliable, practical and have an appropriate impact on student learning” [69]. Perhaps this highlights the need for a standardized, validated instrument to be designed specifically for immersive technology in education for the evaluation of learning outcomes related to the students’ learning experience. For example, in simulation, the National League of Nursing developed a validated standardized scale to evaluate the use of simulation and student learning experience with simulation activity, including students’ satisfaction and self-confidence, perceived learning, and engagement [36]. Therefore, the development of a standardized instrument to evaluate learning outcomes, such as satisfaction, self-confidence, self-efficacy, and engagement, for immersive educational tools may be beneficial so that future research may be better informed by a more uniform approach to assessing learning outcomes.

### Strengths and Limitations

This systematic review provides an up-to-date review on the learning outcomes of immersive technologies in university-level medical and nursing education. This study illustrates important findings on the use of immersive technologies that will provide a foundation for future research in this area. Knowledge gain with immersive technologies was shown in this review to be equal to or greater than that of traditional modalities, thus providing an evidence base for future curriculum developers and researchers alike to implement these immersive tools into university programs. A comprehensive search strategy and robust methodology support the strengths of this study. The use of the validated MERSQI tool to assess the study design of the included studies also adds to the strength of our study design.

Most of the reviewed studies developed their own bespoke immersive educational tool specific to their needs, with VR being primarily used. The favored use of VR may be due in part to the widespread availability of cost-effective 3D software and web-based material for the development of anatomy tools [14,60]. However, the development of these tools has financial and resource implications, including the time spent developing content for these devices. With the increased amount of content and material, there may be an opportunity to develop a universal platform that makes content sharable and available worldwide for health care education. This may reduce the barriers to accepting and implementing this technology in health care education.

Nevertheless, we recognize that there were also some limitations to this study. Common biases exist within the methodology section, including the study eligibility criteria, identification and selection of studies, data extraction, and study appraisal. Predefined search criteria and inclusion criteria were set out in the published protocol, which aimed to reduce these biases. Within the published literature, there is heterogeneity in how VR is defined, requiring us to confine VR to include the use of a headset, providing only an immersive experience, as opposed to a 3D visualization on a computer screen. Therefore, this may have resulted in the exclusion of studies involving a 3D animation or model on a computer screen that did not place the learner in an immersive environment. Furthermore, because of the initial large database of articles retrieved, we decided to only include RCTs, as they are regarded as the highest-quality study type and also included a comparator that was a traditional learning modality. This process was conducted by 2 independent reviewers (GR and SC), and full-text inclusion was dependent on agreement by both reviewers. During the study retrieval process, bias may have occurred because of the unavailability of some studies. Authors were contacted in cases of unavailable data; however, this may have led to data availability bias and unrepresented data. This study included only preclinical university students; therefore, the value of immersive technology in the postqualification setting is unknown.

### Conclusions

In conclusion, this systematic review demonstrates that VR, AR, and MR are beneficial educational tools in preclinical medical and nursing university education. Immersive technology is equally effective in teaching and increases learner satisfaction, self-confidence, and engagement. However, further research is required to explore the role of VR, AR, and MR in midwifery education. With the increasing availability of cost-effective immersive tools, the use of immersive technologies in health care student education is potentially very valuable.

## Appendix

Multimedia Appendix 1 Comprehensive list of search terms developed for the search strategy.

Multimedia Appendix 2 Validated scales used for evaluation.

Multimedia Appendix 3 Medical Education Research Study Quality Instrument domain and item scores for the 29 studies.

## Abbreviations

AR: augmented reality
MERSQI: Medical Education Research Study Quality Instrument
MR: mixed reality
PRISMA: Preferred Reporting Items for Systematic Reviews and Meta-Analyses
PROSPERO: International Prospective Register of Systematic Reviews
RCT: randomized controlled trial
VP: virtual patient
VR: virtual reality
