# Supplementary material for: Learning Outcomes of Immersive Technologies in Health Care Student Education: Systematic Review of the Literature
Source: J Med Internet Res. 2022 Feb 1;24(2):e30082. doi: 10.2196/30082 (PMC8848248; doi:10.2196/30082)
Supplement: Multimedia Appendix 1 [file jmir_v24i2e30082_app1.pdf]

Comprehensive list of search terms developed for the search strategy. The search terms included were divided into four categories: “discipline”, “technology”, “education” and “academic”

Discipline	Technology	Education	Academic
Medical	Virtual Reality	Teaching	Student
Medicine	Augmented Reality	Training	Trainee
Nurse	Mixed Reality	Lecture	
Nursing	Immersive Technology	Pedagogy	
Midwifery	Imaging	Education	
Midwifery	Three dimensional	Learning	
Midwives	Computer Simulation	Tutor	
	Information Technology	Lecturing	
	Educational Technology	Skill	
	High-fidelity Simulation Training		
	Technology		
	Simulation		
	User-computer Interface		
	Computer Simulation		
	Computer Assisted		
