# Supplementary material for: Learning Outcomes of Immersive Technologies in Health Care Student Education: Systematic Review of the Literature
Source: J Med Internet Res. 2022 Feb 1;24(2):e30082. doi: 10.2196/30082 (PMC8848248; doi:10.2196/30082)
Supplement: Multimedia Appendix 2 [file jmir_v24i2e30082_app2.pdf]

Validated scales used for evaluation

Study Title	Validated scale
A Randomised Control Trial and Comparative Analysis of Multi-Dimensional Learning Tools in Anatomy	Cambridge Brain Sciences psychometric tests [33]
Acquisition of Fire Safety Knowledge and Skills with Virtual Reality Simulation	Perioperative Performance evaluation tool [34]
Clinical Virtual Simulation in Nursing Education	Learner Satisfaction with Simulation; General self-efficacy scale [36]
Comparative value of a simulation by gaming and a traditional teaching method to improve clinical reasoning skills necessary to detect patient deterioration: a randomized study in nursing students	Self-assessment of perceived learning [37]
Comparison of virtual patient simulation with mannequin-based simulation for improving clinical performances in assessing and managing clinical deterioration: randomized controlled trial	E-learning system success scale [38]
Finding the Right Blend of Technologically Enhanced Learning Environments: Randomized Controlled Study of the Effect of Instructional Sequences on Interprofessional Learning	Interprofessional Socialisation and valuing scale (ISVS) [41]
HoloLens and mobile augmented reality in medical and health science education: A randomised controlled trial	Adverse health effects (Ames); Participant engagement (McBrien) [42]
Immersive virtual reality as a teaching tool for neuroanatomy	Instructional Materials Motivation Survey [26]
Is individual practice in an immersive and interactive virtual reality application non-inferior to practicing with traditional equipment in learning systematic clinical observation? A randomized controlled trial	System Usability Scale [45]
Learning clinical reasoning: how virtual patient case format and prior knowledge interact	Cognitive load (Opfermann4-item scale) [46]
Mixed reality for teaching catheter placement to medical students: a randomized single-blinded, prospective trial	NASA task load index; Standard system usability scale [47]
Mobile Augmented Reality as a Feature for Self-Oriented, Blended Learning in Medicine: Randomized Controlled Trial	POMS Questionnaire (emotional involvement); AttrakDiff2 Questionnaire [48]
Nurse-Physician Communication Team Training in Virtual Reality Versus Live Simulations: Randomized Controlled Trial on Team Communication and Teamwork Attitudes	ATHCT; ISVS [49]
Problem-based learning in internal medicine: virtual patients or paper-based problems?	Diagnostic Thinking Inventory [51]
The effect of self-practicing systematic clinical observations in a multiplayer, immersive, interactive virtual reality application versus physical equipment: a randomized controlled trial	System Usability Scale [54]
The Effect of Stereoscopic Augmented Reality Visualization on Learning Anatomy and the Modifying Effect of Visual-Spatial Abilities: A Double-Center Randomized Controlled Trial	Mental Rotation test (visual social ability) [55]
The Emotional and Behavioural Impact of Delivering Bad News to Virtual versus Real Standardized Patients: A Pilot Study	Profile of Moods States 2; Maastricht Assessment of Simulated patient [56]
The impact of multi-person virtual reality competitive learning on anatomy education: a randomised controlled study	Intrinsic Motivation Inventory [57]
Virtual reality as a teaching method for resuscitation training in undergraduate first year medical students: a randomized controlled trial	Comparative Self-assessment; System Usability scale [58]
