# Supplementary material for: Learning Outcomes of Immersive Technologies in Health Care Student Education: Systematic Review of the Literature
Source: J Med Internet Res. 2022 Feb 1;24(2):e30082. doi: 10.2196/30082 (PMC8848248; doi:10.2196/30082)
Supplement: Multimedia Appendix 3 [file jmir_v24i2e30082_app3.pdf]

MERSQI domain and item scores for 29 studies

Domain	Item (score)	MERSQI Average Score	SD	Mean (domain)	SD (Domain)	N Studies	%
**Study design**	1. Study design			1	0		
	Randomised controlled trial (3)	3	0			29	100
	Nonrandomised 2 group (2)	0	0			0	0
	Single group pre-test and post-test only (1)	0	0			0	0
**Sampling**	2. No. of institutions studied			0.3	0.15		
	3 institutions (1.5)	0	0			0	0
	2 institutions (1)	0.03	0.2			1	3
	1 institution (0.5)	0.48	0.1			28	97
	3. Response rate %						
	>75% (1.5)	1.4	0.4			27	93
	50-74% (1)	0.07	0.3			2	7
	<50% or not reported (0.5)	0	0			0	0
**Type of data**	4. type of data			0.5	0		
	Assessment by participants (1)	1	0			29	100
	Objective measurement (3)	0	0			0	0
**Validity of evaluation instrument**	5. Internal structure			0.3	0.27		
	Not reported (0)	0	0			12	41
	Reported (1)	0.59	0.5			17	59
	6. Content						
	Not reported (0)	0	0			11	38
	Reported (1)	0.62	0.5			18	62
	7. Relationships to other variables						
	Not reported (0)	0	0			20	69
	Reported (1)	0.31	0.5			9	31
**Data analysis**	8. Appropriateness of analysis			0.7	0.46		
	Appropriate for study design, (1)	1	0			29	100
	Inappropriate for study design or type of data (0)	0	0			0	0
	9. Complexity of analysis						
	Beyond descriptive analysis (2)	1.31	1			19	65
	Descriptive analysis only (1)	0.34	0.5			10	34
**Outcomes**	10. Outcomes			0.6	0.21		
	Satisfaction, attitudes, perceptions, opinions (1)	0.9	0.3			26	90
	Knowledge, Skills (1.5)	1.45	0.3			28	97
	Behaviours (2)	0.14	0.5			2	7
	Patient/health care outcome (3)	0	0			0	0
